# Low GILT Expression is Associated with Poor Patient Survival in Diffuse Large B-Cell Lymphoma

**DOI:** 10.3389/fimmu.2013.00425

**Published:** 2013-12-04

**Authors:** Hannah Phipps-Yonas, Haiyan Cui, Noemi Sebastiao, Patrick S. Brunhoeber, Ellen Haddock, Martin J. Deymier, Wolfram Klapper, Lonnie Lybarger, Denise J. Roe, Karen Taraszka Hastings

**Affiliations:** ^1^Department of Basic Medical Sciences, University of Arizona, Phoenix, AZ, USA; ^2^Arizona Cancer Center, University of Arizona, Tucson, AZ, USA; ^3^Ventana Medical Systems, Tucson, AZ, USA; ^4^Department of Cellular and Molecular Medicine, University of Arizona, Tucson, AZ, USA; ^5^Institute of Pathology, Hematopathology Section and Lymph Node Registry, Universitätsklinikum Schleswig-Holstein, Kiel, Germany; ^6^Department of Immunobiology, University of Arizona, Tucson, AZ, USA; ^7^Mel and Enid Zuckerman College of Public Health, University of Arizona, Tucson, AZ, USA

**Keywords:** GILT, MHC class II, antigen processing and presentation, diffuse large B cell lymphoma, tumor immunology

## Abstract

The major histocompatibility complex (MHC) class II-restricted antigen processing pathway presents antigenic peptides acquired in the endocytic route for the activation of CD4^+^ T cells. Multiple cancers express MHC class II, which may influence the anti-tumor immune response and patient outcome. Low MHC class II expression is associated with poor survival in diffuse large B-cell lymphoma (DLBCL), the most common form of aggressive non-Hodgkin lymphoma. Therefore, we investigated whether gamma-interferon-inducible lysosomal thiol reductase (GILT), an upstream component of the MHC class II-restricted antigen processing pathway that is not regulated by the transcription factor class II transactivator, may be important in DLBCL biology. GILT reduces protein disulfide bonds in the endocytic compartment, exposing additional epitopes for binding to MHC class II and facilitating antigen presentation. In each of four independent gene expression profiling cohorts with a total of 585 DLBCL patients, low GILT expression was significantly associated with poor overall survival. In contrast, low expression of a classical MHC class II gene, HLA-DRA, was associated with poor survival in one of four cohorts. The association of low GILT expression with poor survival was independent of established clinical and molecular prognostic factors, the International Prognostic Index and the cell of origin classification, respectively. Immunohistochemical analysis of GILT expression in 96 DLBCL cases demonstrated variation in GILT protein expression within tumor cells which correlated strongly with GILT mRNA expression. These studies identify a novel association between GILT expression and clinical outcome in lymphoma. Our findings underscore the role of antigen processing in DLBCL and suggest that molecules targeting this pathway warrant investigation as potential therapeutics.

## Introduction

The major histocompatibility complex (MHC) class II-restricted antigen processing pathway generates cell-surface peptide-MHC class II complexes essential for the activation of CD4^+^ T cells (1). The three classical MHC class II proteins, HLA-DP, HLA-DQ, and HLA-DR, are heterodimers composed of an α and β chain encoded by A and B genes, respectively (e.g., HLA-DRA and HLA-DRB) (2). MHC class II molecules assemble with invariant chain (Ii) in the endoplasmic reticulum. Ii is responsible for trafficking the class II-Ii complex to the endocytic pathway and protecting the peptide binding groove from prematurely acquiring a peptide. Gamma-interferon-inducible lysosomal thiol reductase (GILT) catalyzes the reduction of protein disulfide bonds in the late endosomes and lysosomes, thereby exposing buried peptide epitopes for binding to MHC class II and enhancing the MHC class II-restricted presentation of a number of epitopes (3–9). In the lysosomes, proteases degrade endocytosed and endogenous proteins to form class II-binding peptides. A non-classical MHC class II protein HLA-DM mediates loading MHC class II with a high affinity peptide. Peptide-MHC class II complexes are then directed to the cell surface for recognition by and activation of CD4^+^ T cells. CD4^+^ T cells augment anti-tumor immunity, in part through promoting the differentiation and maintenance of CD8^+^ cytotoxic T cells (10–12). MHC class II molecules are expressed by professional antigen presenting cells (APCs), such as dendritic cells (DCs) and B cells, as well as cancers derived from these cell types. In addition, ectopic expression of MHC class II has been described on multiple cancers, including melanoma, breast, colon, thyroid, and cervical (6, 13–16). Therefore, MHC class II-restricted processing and presentation by tumor cells may exert a major influence over T cell responses and play a critical role in anti-tumor immunity.

Diffuse large B-cell lymphoma (DLBCL) is the most common type of aggressive lymphoid neoplasm, accounting for 30% of adult non-Hodgkin lymphomas (17). DLBCL is a heterogeneous disease with large variations in clinical outcome (17). Prognostic stratification is based on clinical risk factors used to determine the International Prognostic Index (IPI), which incorporates patient age at diagnosis, tumor stage, serum lactate dehydrogenase level, performance status, and number of extra-nodal sites (18). The combination of cyclophosphamide, hydroxydaunorubicin (doxorubicin), Oncovin^®^(vincristine), and prednisone (CHOP) had been the standard chemotherapy regimen for decades. The recent addition of rituximab, a monoclonal antibody (mAb) to CD20, to the treatment protocol (R-CHOP) has significantly improved patient survival (19, 20). Gene expression profiling (GEP) studies have demonstrated molecular heterogeneity in histologically similar DLBCL tumors and identified gene expression signatures which could offer more accurate prognostic stratification compared to the IPI (21–29). Alizadeh et al. demonstrated distinct molecular subgroups based on the cell of origin (COO) or differentiation state of the tumor (21). Patients with germinal center B cell-like (GCB) DLBCL have significantly improved survival compared to activated B cell-like (ABC) DLBCL (21). Subsequent GEP studies have identified that the immune response plays a favorable role in prognosis (22, 27, 28). In particular, low expression of genes involved in MHC class II-restricted antigen presentation is associated with poor survival in DLBCL (22, 27, 30–32).

Gamma-interferon-inducible lysosomal thiol reductase is a key component of the MHC class II-restricted antigen processing and presentation pathway. However, unlike other members of this pathway including HLA-DR, HLA-DP, HLA-DQ, HLA-DM, and li, GILT is not regulated by transcription factor class II transactivator (CIITA) (33–35). Therefore, we sought to determine whether GILT expression levels may provide further prognostic value. Although GILT mRNA expression was previously identified as part of the “host-response signature” in DLBCL by Monti et al. this study did not observe an association of the “host-response signature” with survival (26). We hypothesized that low GILT expression, which is predicted to result in impaired presentation of a subset of class II-restricted epitopes, would be associated with poor patient survival. In each of four independent GEP cohorts with a total of 585 DLBCL patients, low GILT expression was associated with poor overall survival. GILT was a better predictor of survival than HLA-DRA, a classical MHC class II gene. GILT expression was independent of the clinical IPI score, and the association of GILT expression with survival was independent of COO classification, suggesting that GILT represents an important independent aspect of tumor biology. Immunohistochemical analysis of GILT expression in 96 DLBCL tissue microarray (TMA) specimens demonstrated that variation in GILT protein expression within tumor cells correlated with GILT mRNA expression from the GEP studies. This study demonstrates that GILT expression levels correlate with clinical outcome and underscore that the antigen processing pathway is critical to patient survival in DLBCL.

## Materials and Methods

### Patient characteristics and GEP datasets

Using the National Center for Biotechnology Information (NCBI) Gene Expression Omnibus (GEO), we identified three GEP datasets from pre-treatment DLBCL biopsy specimens that included patient survival data and probes for quantification of GILT and HLA-DRA mRNA expression (Lenz: http://www.ncbi.nlm.nih.gov/geo/query/acc.cgi?acc=GSE10846, Hummel: http://www.ncbi.nlm.nih.gov/geo/query/acc.cgi?acc=GSE4475, Shaknovich: http://www.ncbi.nlm.nih.gov/geo/query/acc.cgi?acc=GSE23501, Table 1). Data regarding overall survival, follow-up time, IPI, COO classification, and morphologic and molecular diagnosis were collected directly from the GSE file. The Lenz et al. dataset was divided into two groups based on treatment regimen (CHOP vs. R-CHOP) (23). The Hummel et al. dataset contained 220 mature aggressive B-cell lymphomas; we included all patients who had survival information and the morphologic diagnosis of DLBCL, but did not have the molecular diagnosis of Burkitt lymphoma and did not receive rituximab (24). All patients in the Shaknovich et al. dataset were included in our analysis (29). Gene probes were identified based on reported platform data. GILT expression was represented by the single probe ID 201422_at. HLA-DRA expression was represented by two probes: IDs 208894_at and 210982_s_at.

**Table 1:** **Summary of DLBCL GEP cohorts**.

Cohort and treatment	Lenz CHOP	Hummel CHOP	Lenz R-CHOP	Shaknovich R-CHOP
No. of patients	181	102	233	69
Study group	Multinational LLMPP	Multinational MMMLNP	Multinational LLMPP	British Columbia Cancer Agency
End points and covariates	OS, IPI, COO	OS, COO	OS, IPI, COO	OS, PFS, IPI, COO
Median age, years (range)	62 (14–88)	65 (8–93)	60 (17–92)	62 (16–92)
IPI score (%)
IPI = 0	18/157 (11)	N/A	28/164 (17)	0/65 (0)
IPI = 1	44/157 (28)		42/164 (26)	12/65 (18)
IPI = 2	53/157 (34)		43/164 (26)	21/65 (32)
IPI = 3	29/157 (18)		30/164 (18)	19/65 (29)
IPI = 4	13/157 (8)		16/164 (10)	13/65 (20)
IPI = 5	0/157 (0)		5/164 (3)	0/65 (0)
COO (%)
ABC-like	74/181 (41)	37/102 (36)	93/233 (40)	20/69 (29)
GCB-like	76/181 (42)	38/102 (37)	107/233 (46)	40/69 (58)
Unclassified	31/181 (17)	27/102 (26)	33/233 (14)	9/69 (13)
Primary data source	NCBI GSE10846	NCBI GSE4475	NCBI GSE10846	NCBI GSE23501
Measurement platform	Affymetrix-HG U133 Plus 2.0	Affymetrix-HG U133A	Affymetrix-HG U133 Plus 2.0	Affymetrix-HG U133 Plus 2.0

*LLMP, Lymphoma and Leukemia Molecular Profiling Project; MMMLNP, Molecular Mechanisms in Malignant Lymphomas Network Project of the Deutsche Krebshilfe, OS, overall survival; PFS, progression free survival; IPI, international prognostic index; N/A, not available; COO, cell of origin classification; ABC, activated B cell-like; GCB, germinal center B cell-like; NCBI, National Center of Biotechnology Information; GSE, gene expression omnibus series. The number of patients in each cohort indicates the total number of patients with DLBCL with OS data. Since the IPI score and COO were not reported for all patients, the *n* value is decreased in the IPI and COO analyses. References for the cohorts are (23, 24, 29)*.

A cut-off point of the 10% of patients with the lowest gene expression was selected *a priori* and used to examine the association with survival. For GILT, this cut-off point was selected based on the enzymatic activity of GILT, as a loss of GILT function is only anticipated with a significant loss or absence of GILT expression. This concept has been validated *in vivo*, as we observe a phenotypic difference between wild-type and GILT knockout mice, but not between wild-type and GILT heterozygous mice (4). The cut-off point of 10% was selected for HLA-DRA, because prior assessment of multiple cut-off points demonstrated that the 10% of patients with the lowest HLA-DRA gene expression have the highest risk of death and worst survival in DLBCL and that the hazard ratio (HR) of death is a smooth, non-linear function of HLA-DRA expression (27). Therefore, we used the previously determined cut-off point for HLA-DRA expression in DLBCL survival.

### Statistical analysis

The correlation between two HLA-DRA probes was tested using the Spearman correlation coefficient. Since this correlation was statistically significant, the average expression value from the two probes was used in the analysis. The association between survival and gene expression was assessed by comparing the survival for ≤10 vs. >10% GILT and HLA-DRA expression levels. This cut point was selected based on the rationale as discussed above. Subsequently, alternative cut-off points (20, 30, and 50%) were explored. Only the 10% cut-off point led to a statistically significant relationship between low GILT expression and poor survival across all four cohorts (data not shown). When HLA-DRA was assessed with multiple cut-off points, a significant relationship between low HLA-DRA and survival was only observed in the Lenz CHOP cohort (data not shown). Since the additional cut-off points did not lead to significant results across the four cohorts as expected, we focused on the presentation of the analysis with the 10% cut-off. The non-parametric Kaplan–Meier method was used to estimate the overall survival curve for each group. A Cox proportional hazards model was used to test the difference in overall survival between the groups using the *p*-value from the score test; the HR and its 95% confidence interval (CI) also were reported. Additional analyses assessed the association between survival and GILT expression adjusted for COO using the Cox proportional hazards model. For the association between GILT expression and IPI score, analysis of variance was employed. A two sample independent *t*-test was used to compare GILT expression in GCB and ABC subtypes. The Spearman correlation coefficient was used to test the correlation between GILT and HLA-DRA expression, and GILT mRNA and protein expression. A *p*-value <0.05 was considered significant. Analyses were conducted using SAS V9.1.2 software (Cary, NC, USA).

### Cells and tissues

De-identified formalin-fixed paraffin-embedded tonsil tissue blocks were obtained from the Research and Development Tissue Bank at Ventana Medical Systems (Tucson, AZ, USA). Formalin-fixed paraffin-embedded TMAs were generously provided by Dr. Wolfram Klapper and the Molecular Mechanisms in Malignant Lymphomas Network Project (MMMLNP). The human subjects protocols of the MMMLNP were approved by the ethics committees of all participating institutions (24). The TMAs contained 47 tumor sections from the Hummel CHOP cohort and an additional 49 DLBCL specimens from GEO database GSE22470 (36, 37), which were selected by the same criteria as the Hummel CHOP cohort but did not contain survival data.

### Immunohistochemistry

Immunohistochemical staining was performed on a Benchmark ULTRA instrument (Ventana Medical Systems). Heat-induced epitope retrieval was performed using Cell Conditioning-1 (Ventana Medical Systems). Sections were blocked using BLOXALL™ endogenous peroxidase and alkaline phosphatase blocking solution (Vector Laboratories, Burlingame, CA, USA). Sections were stained with rabbit anti-GILT polyclonal antibody (Catalog# S1265, 1:3000 dilution, Epitomics, Burlingame, CA, USA) followed by the OptiView DAB IHC detection kit, Hematoxylin II counterstain, and Bluing reagent (Ventana Medical Systems). Universal negative control serum (BioCare Medical, LLC, Concord, CA, USA) served as a negative control. DLBCL cell lines OCI-LY3, which is of the ABC subgroup, and OCI-LY19, which is of the GCB subgroup, were provided by the Arizona Lymphoid Tissue and Blood Repository (University of Arizona, Tucson, AZ, USA) (21, 38).

Staining of the TMA cores was scored with light microscopy by a board-certified pathologist blinded to data associated with the specimens. The DLBCL specimens were evaluated for the predominant tumor cell staining intensity, percent of tumor cells staining, predominant APC staining intensity, and percent of APCs present in the tumor. Staining was scored using a scale from 0 to 4 with 0.25 increments based on the intensity of the staining and the amount of stained vesicles within each cell. The predominant staining intensity present in the cells of interest was used.

### Confocal microscopy

Lymphoma cell lines were plated on alcian blue-coated coverslips, fixed with acetone, and permeabilized with methanol. Antibodies were diluted in PBS with 0.05% saponin, 10% FBS, 10 mM glycine, 10 mM HEPES pH 7.4, and 0.5% sodium azide. Cells were stained with rabbit anti-human GILT serum (1:800 dilution; kindly provided by Dr. Peter Cresswell) and mouse anti-human HLA-DR mAb (2.5 μg/ml, clone G46-6, BD Pharmingen, San Jose, CA, USA). Secondary antibodies Alexa Fluor 555-conjugated goat anti-rabbit IgG (H + L) and Alexa Fluor 488-conjugated goat anti-mouse IgG (H + L) (Invitrogen, Carlsbad, CA, USA) were used at 2.5 μg/ml. Nuclei were detected with Hoechst 33342 (1:15,000 dilution; Invitrogen).

## Results

### Variation in GILT expression in DLBCL and cut point determination

**Figure 1:**
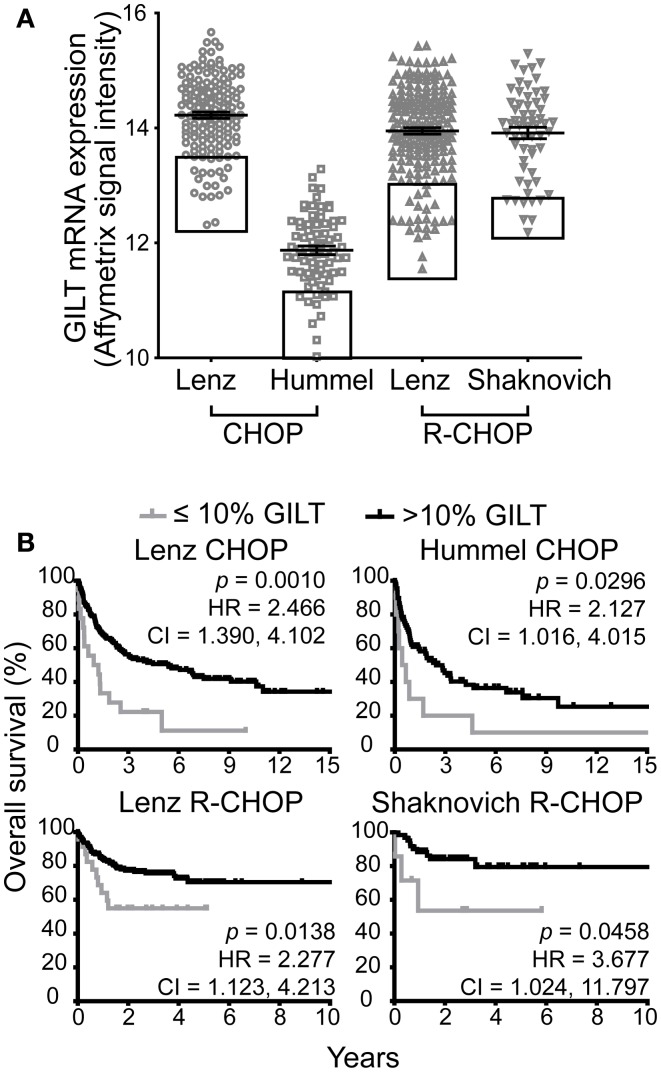
**Low GILT gene expression is associated with poor survival in all DBLCL cohorts**. **(A)** The relative mRNA expression of GILT in each patient in the four cohorts. Each symbol represents a single patient. The line and error bars indicate the mean ± SEM. The box indicates the 10% of patients with the lowest GILT expression. **(B)** Kaplan–Meier survival curves comparing patients with ≤10 (gray line) vs. >10% GILT gene expression (black line). The *p*-values, hazard ratio (HR), and 95% confidence interval (CI) comparing survival of patients with ≤10 and >10% expression of GILT are indicated for each cohort. Lenz CHOP: *n* = 18 ≤ 10% GILT, *n* = 163 > 10% GILT, Hummel CHOP: *n* = 10 ≤ 10% GILT, *n* = 92 > 10% GILT, Lenz R-CHOP: *n* = 23 ≤ 10% GILT, *n* = 210 > 10% GILT, Shaknovich R-CHOP: *n* = 7 ≤ 10% GILT, *n* = 62 > 10% GILT.

We identified four DLBCL cohorts (Table 1) to examine the relationship between GILT expression in DLBCL tumors and overall patient survival. Figure 1A illustrates the range of GILT mRNA expression in DLBCL specimens from patients in the four cohorts. These data show that all cohorts exhibited variation in GILT expression in DLBCL tumors across patients. The shift in relative GILT expression in the Hummel et al. cohort is due to a difference in measurement platform. Boxes depict the 10% of patients with the lowest GILT expression who were selected to examine the impact of low GILT expression on overall survival in DLBCL (Figure 1A).

The cut-off point of the 10% of patients with the lowest GILT gene expression was selected *a priori* prior to evaluation of the datasets. Taking into account that GILT is an enzyme which catalyzes multiple rounds of reactions, a loss in function is only anticipated in the patients with the lowest levels of gene expression. Supporting the hypothesis, we have previously reported that while there is a significant difference in MHC class II-restricted T cell responses between GILT knockout and wild-type mice, no difference in phenotype is observed between GILT heterozygous and wild-type mice (4). Therefore, we utilized the ≤10% GILT expression cut-off point to represent the patients with a significant loss or absence of GILT.

### Low GILT expression is associated with poor survival in all DLBCL cohorts

We compared the Kaplan–Meier survival curves of patients with ≤10 vs. >10% GILT mRNA expression in the pre-treatment tumor biopsy (Figure 1B). In each cohort, patients with low GILT expression had significantly shortened overall survival compared to patients with higher GILT expression. In the Lenz CHOP cohort, the median overall survival of patients with low GILT expression was 1.1 years compared to 5.4 years with high GILT expression. In the Hummel CHOP cohort, the median survival significantly increased from 0.6 years in patients with the lowest 10% GILT expression to 2.5 years in the remaining patients. Median survival time could not be determined in the two R-CHOP cohorts as survival was significantly improved and the majority of patients survived during the follow-up period. Despite the improved survival in patients treated with rituximab, low GILT expression remained significantly associated with poor survival in the Lenz R-CHOP and Shaknovich R-CHOP cohorts (*p* = 0.014 and *p* = 0.046, respectively). To further quantify the impact of low GILT expression on survival in DLBCL, the HR and 95% CIs were determined for all cohorts comparing the 10% of patients with the lowest GILT expression with the remaining 90% of patients (Figure 1B). Patients with low GILT expression had a 2.1–3.7 times greater risk of death compared to patients with higher GILT expression. These data show that patients with low GILT expression in the DLBCL tumor have significantly poorer overall survival.

### Low HLA-DRA expression is associated with poor survival in one of four cohorts

**Figure 2:**
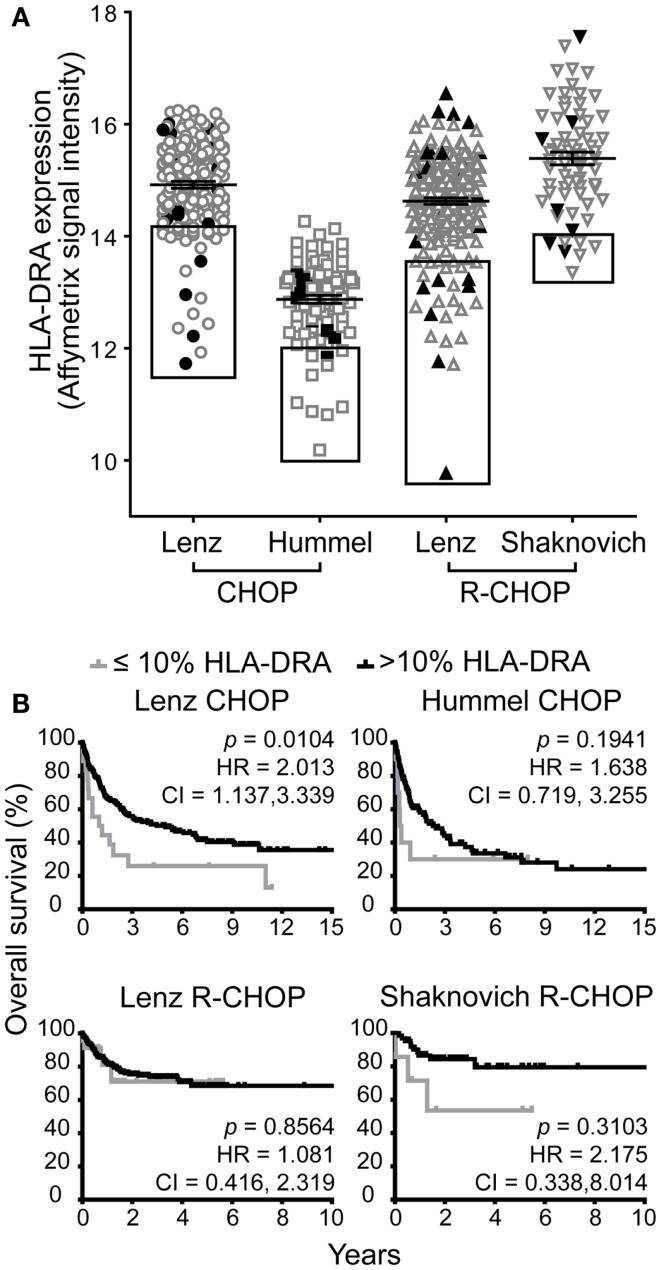
**Low HLA-DRA gene expression is associated with poor survival in one of four DBLCL cohorts**. **(A)** The relative mRNA expression of HLA-DRA in each patient in the four cohorts. Each symbol represents a single patient. The line and error bars indicate the mean ± SEM. The box indicates the 10% of patients with the lowest HLA-DRA expression. The closed, black symbols denote the 10% of patients with the lowest GILT expression shown in the boxes in Figure 1A. **(B)** Kaplan–Meier survival curves comparing patients with ≤10% expression of HLA-DRA (gray line) to patients with >10% expression (black line). The *p*-values, hazard ratio (HR), and 95% confidence interval (CI) comparing survival of patients with ≤10 and >10% expression of HLA-DRA are indicated for each cohort. Lenz CHOP: *n* = 18 ≤ 10% HLA-DRA, *n* = 163 > 10% HLA-DRA, Hummel CHOP: *n* = 10 ≤ 10% HLA-DRA, *n* = 92 > 10% HLA-DRA, Lenz R-CHOP: *n* = 23 ≤ 10% HLA-DRA, *n* = 210 > 10% HLA-DRA, Shaknovich R-CHOP: *n* = 7 ≤ 10% HLA-DRA, *n* = 62 > 10% HLA-DRA.

Next, we compared the association of low GILT gene expression with poor survival to the impact of low expression of a representative MHC class II gene HLA-DRA. Low HLA-DR gene and protein expression have previously been shown to be associated with poor survival (22, 27, 30–32, 39–41). Prior studies have demonstrated that the HR of death in DLBCL is a smooth, non-linear function of HLA-DRA expression and that the 10% of patients with the lowest HLA-DRA gene expression have the highest risk of death and worst survival (27). Therefore, we used this established cut-off point to evaluate the effect of HLA-DRA expression on DLBCL survival in the four cohorts. Figure 2A demonstrates the range in HLA-DRA mRNA expression in DLBCL specimens from patients in the four cohorts. All cohorts exhibited variation in HLA-DRA expression in DLBCL tumors across patients; as in Figure 1A, the relative shift in expression in the Hummel et al. cohort is due to a difference in measurement platform. Boxes depict the 10% of patients with the lowest HLA-DRA expression who were selected to examine the impact of low HLA-DRA expression on overall survival in DLBCL (Figure 2A). The closed black symbols in Figure 2A denote the 10% of patients with the lowest GILT expression, demonstrating that the 10% of patients with the lowest GILT expression are not the same as the 10% of patients with the lowest HLA-DRA expression. To further explain this difference, we analyzed the correlation between GILT and HLA-DRA expression. We found that GILT expression moderately correlated with HLA-DRA expression in both CHOP cohorts (Lenz CHOP, Spearman ρ = 0.39, *p* < 0.0001; Hummel CHOP, Spearman ρ = 0.31, *p* = 0.0016), and GILT expression did not correlate with HLA-DRA in either of the R-CHOP cohorts (Lenz R-CHOP, Spearman ρ = 0.068, *p* = 0.30; Shaknovich R-CHOP, Spearman ρ = 0.089, *p* = 0.47). This is in contrast to the strong correlation between HLA-DRA expression and MHC class II-restricted antigen processing pathway members also regulated by CIITA. The correlation of HLA-DRA expression with expression of HLA-DRB, HLA-DPA, HLA-DPB, HLA-DQA, HLA-DQB, HLA-DMA, HLA-DMB, and Ii has previously been reported with Pearson correlation coefficients between 0.85 and 0.92 (*p* < 0.001) (27).

Low HLA-DRA expression was significantly associated with poor survival in the Lenz CHOP group (*p* = 0.010), but not in the other three cohorts (Figure 2B). Similarly, a significant two-times increased hazard of death with low HLA-DRA expression was identified in the Lenz CHOP cohort, but not the other cohorts (Figure 2B). This result is consistent with the loss of the association between improved survival and the “MHC class II” signature in the Lenz R-CHOP cohort (23). Together these data show that while low GILT gene expression is associated with poor survival in all CHOP and R-CHOP cohorts, low HLA-DRA gene expression is only associated with poor survival in a single cohort.

### GILT expression is not associated with the IPI

Patient prognosis in DLBCL is predicted by clinical factors that comprise the IPI. The IPI ranges from 0 to 5, with each of the five criteria receiving a point for an unfavorable value. We sought to determine whether GILT expression is associated with the IPI score. The IPI score for the Hummel CHOP cohort could not be determined as patient data for three criteria were not reported. For the three cohorts examined, there were no statistically significant differences in the mean GILT expression levels across the IPI scores (Table 2) (*p* > 0.65 in each). These data show that GILT expression is not associated with IPI.

**Table 2:** **GILT expression is not associated with IPI score**.

	Mean GILT mRNA expression (SE)						*p*-value
	IPI = 0	IPI = 1	IPI = 2	IPI = 3	IPI = 4	IPI = 5	
Lenz CHOP	14.37 (0.11)	14.17 (0.09)	14.21 (0.10)	14.13 (0.15)	14.04 (0.18)	NA	0.6842
Lenz R-CHOP	14.04 (0.13)	13.96 (0.11)	13.96 (0.13)	13.88 (0.17)	13.69 (0.21)	13.72 (0.42)	0.7752
Shaknovich R-CHOP	NA	13.91 (0.18)	13.84 (0.20)	13.84 (0.18)	14.05 (0.24)	NA	0.8796

*NA indicates not applicable as no patients fit the criteria*.

### Association of GILT expression with survival is independent of COO classification

**Figure 3:**
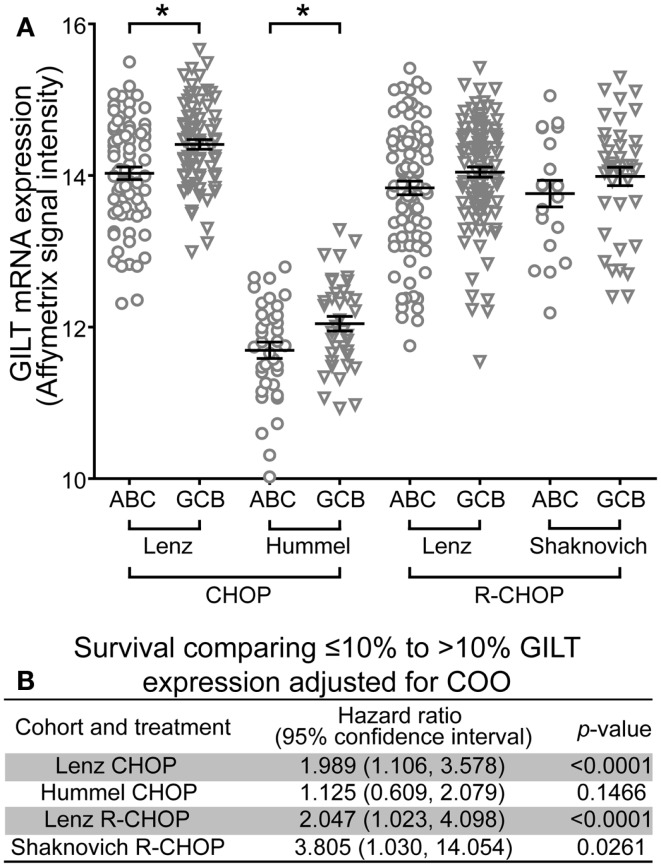
**Association of GILT expression with survival is independent of COO classification**. **(A)** The relative mRNA expression of GILT in patients classified as GCB or ABC subtype in the four cohorts. Each symbol represents an individual patient. Comparison of GILT expression in GCB and ABC subtypes using a two sample independent *t*-test revealed *p* = 0.0003 in the Lenz CHOP, *p* = 0.0179 in the Hummel CHOP, *p* = 0.0653 in the Lenz R-CHOP, and *p* = 0.2887 in the Shaknovich R-CHOP cohort. The line and error bars indicate the mean ± SEM (*p < 0.05). **(B)** Adjusted for COO subtype, the hazard ratio, 95% confidence interval and *p*-value comparing survival of patients with ≤10 to >10% expression of GILT in each cohort.

The COO classification scheme differentiates DLBCL into ABC and GCB subgroups (21). The ABC subtype correlates with poor prognosis (21, 22, 42, 43). To determine whether GILT expression was higher in a particular subgroup, relative GILT expression was plotted for each patient that was classified into ABC or GCB subgroups in Table 1 (Figure 3A). In each cohort, the mean GILT expression was greater in patients in the GCB subgroup compared to the ABC subgroup. The increase in GILT expression was statistically significant in the Lenz and Hummel CHOP cohorts (*p* = 0.0003 and *p* = 0.018, respectively). To investigate whether the impact of GILT expression on survival is independent of COO classification, we determined the HR comparing ≤10 and >10% GILT expression adjusting for COO. Low GILT expression conferred a two-times or greater increased risk of death independent of COO in three cohorts (Lenz CHOP, Lenz R-CHOP, and Shaknovich R-CHOP) (Figure 3B). In the fourth cohort, Hummel CHOP, the HR was no longer statistically significant when adjusted for COO (Figure 3B). Overall, these data strongly support that the association of GILT expression with survival in DBLCL is independent of COO classification.

### GILT protein is expressed in B cells and APCs in benign lymphoid tissue

**Figure 4:**
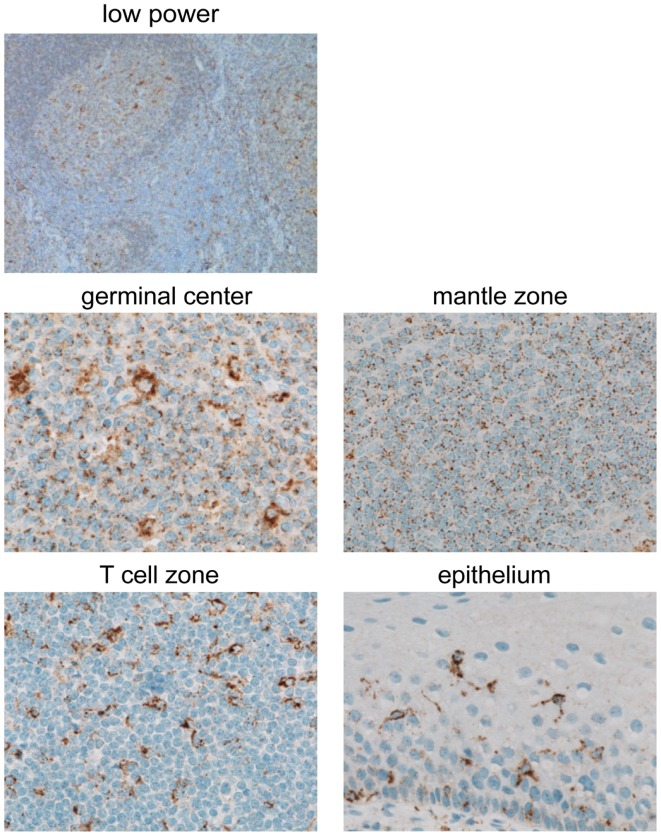
**GILT protein is expressed in B cells and APCs in benign tonsil**. GILT expression in normal human tonsil shown with a low power view (original magnification, ×100) and in the germinal center, mantle zone, T cell zone, and epithelium (original magnification, ×600). Images were acquired using a Nikon ECLIPSE 80i microscope with a 10×/0.30 or 60×/0.95 objective lenses, a Nikon DS-Ri1 camera and Nikon NIS-Elements Imaging software v3.13.

Although GILT is known to be constitutively expressed in many APC types including B cells and DCs (3–6), to our knowledge, GILT expression in normal lymphoid organs has not been reported. We examined GILT protein expression in tonsil by immunohistochemistry. Figure 4 shows a low power view of staining with an anti-GILT polyclonal antibody demonstrating GILT expression within the germinal center, mantle zone, and T cell zone. Upon higher magnification, GILT staining was observed within the B cells and follicular DCs of the germinal center, naïve B cells in the mantle zone, interdigitating DCs in the T cell zone, and Langerhans cells in the tonsillar epithelium (Figure 4). Consistent with GILT’s known localization to lysosomes and late endosomes, GILT staining revealed a punctate intracellular pattern in B cells and APCs of the tonsil (Figure 4). Of note, GILT staining was not detected in T cells, epithelial cells, muscle cells, or salivary glands (Figure 4 and data not shown).

### GILT protein expression in DLBCL

**Figure 5:**
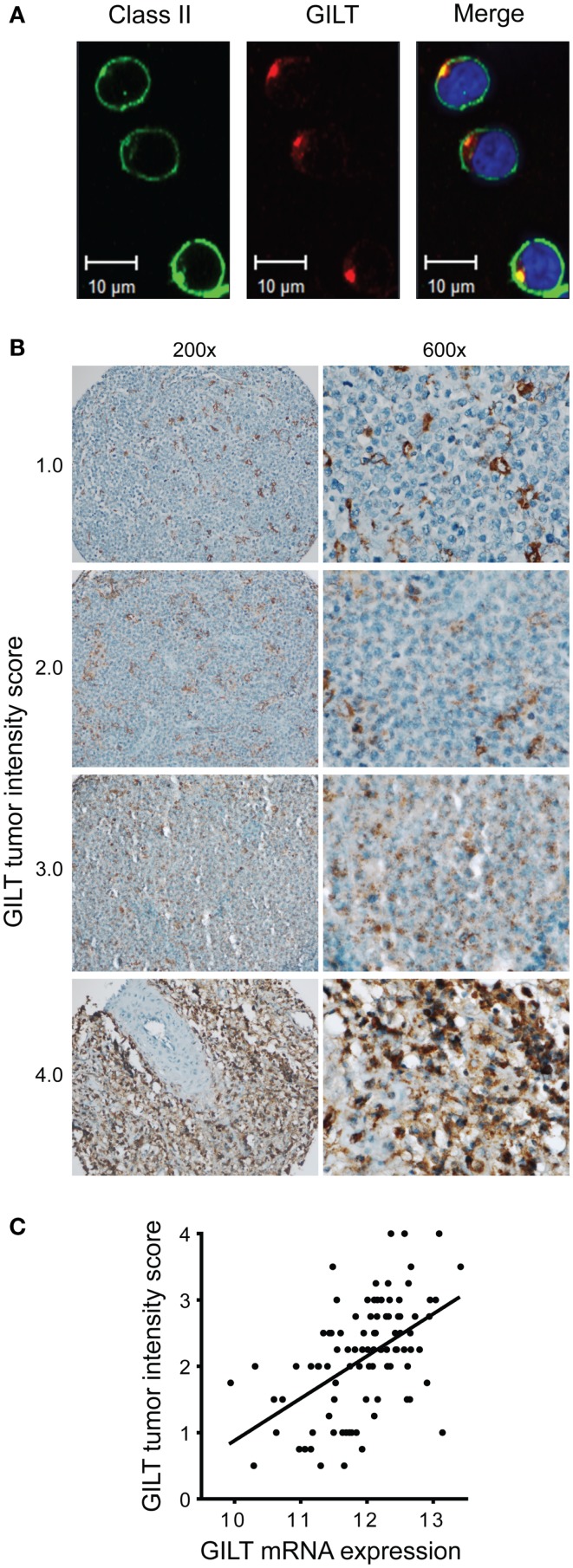
**Variation in GILT protein expression in DLBCL tumor cells correlates with mRNA expression**. **(A)** In OCI-LY19, a DLBCL GCB subtype cell line, GILT and HLA-DR expression were detected with Alexa Fluor 555-conjugated and Alex Flour 488-conjugated secondary antibodies, respectively. Images were viewed using a Zeiss LSM 710 confocal microscope with a Plan-APOCHROMAT 63×/1.4 objective lens and Immersol 518, and acquired with Zen LE software (Carl Zeiss Microimaging Inc., Thornwood, NY, USA). **(B)** Low (original magnification, ×200) and high power view (original magnification, ×600) of representative GILT staining in DLBCL patient TMA specimens. Each specimen is labeled with GILT tumor intensity score, which reflects the intensity of GILT staining and the number of GILT-expressing vesicles in the malignant B cells (scale from 0 to 4). As a reference, benign tonsillar B cells were scored as 2.0. Images were acquired using a Nikon ECLIPSE 80i microscope with a 20×/0.50 or 60×/0.95 objective lenses, a Nikon DS-Ri1 camera and Nikon NIS-Elements Imaging software v3.13. **(C)** GILT mRNA expression in the tumors from 96 DLBCL patients in GEP studies correlated with GILT protein expression in the tumor cells (GILT tumor intensity score) (Spearman ρ = 0.53, *p* < 0.0001).

Next, we determined which cell types in DLBCL tumors express GILT and in which cell type GILT expression varies and thus may impact tumor biology. We began by evaluating GILT protein expression in DLBCL cell lines OCI-LY19 (GCB subgroup) and OCI-LY3 (ABC subgroup) by confocal microscopy. Similar to immunohistochemistry, confocal microscopy revealed a punctate pattern of GILT staining and a predominantly cell-surface pattern together with a punctate pattern of MHC class II expression in DLBCL cell lines (Figure 5A and data not shown). GILT and MHC class II expression in DLBCL cell lines colocalized in the intracellular MHC class II loading compartment (Figure 5A and data not shown). These findings demonstrated that DLBCL tumors cells express GILT and that GILT maintains the same intracellular localization as in benign B cells.

Then, we examined GILT protein expression in 96 DLBCL tissue specimens in a TMA. A previous study identified GILT protein expression in tumor-infiltrating DCs in DLBCL, but did not report GILT expression in tumor cells (26). By using either a polyclonal antibody (as in Figure 4) or a mAb with sensitive immunohistochemical techniques including amplification steps (data not shown), we detected GILT expression in both malignant B cells and APCs in DLBCL tumors (Figure 5B). Our results are supported by GILT expression in DLBCL cell lines OCI-LY3 and OCI-LY19 (Figure 5A) and are in agreement with other studies showing GILT is constitutively expressed by primary B cells and B cell lymphoma lines (3–6). As in the confocal microscopy of DLBCL cell lines and immunohistochemistry of benign B cells and other APCs, we observed a punctate pattern of GILT staining in the malignant B cells (Figure 5B). GILT staining was scored on a scale from 0 to 4 based on the staining intensity and number of GILT-expressing vesicles per cell. There was significant variation in GILT protein expression in malignant B cells reflected by differences in the tumor intensity score (Figure 5B). Within DLBCL cells, the range of GILT expression was from 0.5 to 4 with a mean tumor intensity score of 2.13 ± 0.8 and a mean frequency of 69% ± 18%. In contrast, GILT expression was uniformly high in tumor-infiltrating APCs (mean intensity score = 3.65 ± 0.55, mean frequency within tumor = 12% ± 11%). Even in cases with low GILT expression in tumor cells, tumor-infiltrating APCs were strongly positive (Figure 5B). GILT mRNA expression from GEP studies correlated with the GILT tumor intensity score (Spearman ρ = 0.53, *p* < 0.0001) (Figure 5C). There was no statistically significant correlation between GILT mRNA and protein expression in tumor-infiltrating APCs (data not shown). These data definitively demonstrate variation in GILT protein expression in DLBCL tumor cells which correlates with GILT mRNA expression that is associated with patient survival.

## Discussion

We evaluated the impact of expression of a key enzyme involved in antigen processing on patient survival in DLBCL. Low GILT gene expression, which is anticipated to correlate with a lack of GILT protein function, was significantly associated with poor survival in four patient cohorts, including patients treated with R-CHOP. Furthermore, GILT expression was not associated with the clinical IPI score, and the association of GILT expression with survival was independent of COO classification. Thus, GILT may relate to an important independent aspect of tumor biology.

GILT functions in the MHC class II-restricted antigen processing pathway by reducing disulfide bonds in the endocytic compartment to expose buried epitopes for class II-binding. GILT has been shown to enhance MHC class II-restricted presentation of multiple epitopes from disulfide bond-containing antigens, including the model antigen hen egg lysozyme (3), tumor antigens tyrosinase and tyrosinase-related protein 1 (4, 6), viral glycoproteins (7), autoantigen myelin oligodendrocyte glycoprotein (8), and dust mite allergen Der p 1 (9). Low or absent GILT expression in MHC class II-expressing melanoma cell lines results in diminished MHC class II-restricted presentation of endogenous tumor antigens (6). Furthermore, deficient GILT expression diminishes CD4^+^ T cell-mediated responses in *in vivo* mouse models (3, 4, 9). In B cell lymphoma lines, manipulations that increase MHC class II and GILT expression improve CD4^+^ T cell recognition (44). Therefore, loss of GILT expression in DLBCL cells is anticipated to diminish MHC class II-restricted presentation of tumor antigens and decrease anti-tumor T cell responses.

The association of low GILT expression with survival is stronger than the association of a classical MHC class II gene with survival. While the negative consequence of low gene and protein expression of MHC class II molecules on patient survival in DLBCL is well-documented, the significance of low HLA-DR expression on survival has varied between studies and when different parameters were used (22, 23, 27, 30–32, 39–41). Our data suggest that the quality of antigen processing (i.e., the ability to generate tumor-specific epitopes) is more critical to patient survival than the abundance of a particular MHC class II molecule for antigen presentation. As an upstream member in the MHC class II-restricted antigen processing and presentation pathway, a change in GILT expression has the ability to alter the epitopes that are presented and shape the T cell response. In addition, GILT expression may be a stronger predictor of survival than any single class II gene, because GILT’s reductase activity is anticipated to improve presentation by HLA-DR, HLA-DP, and HLA-DQ. Though, in general, expression of these classical class II genes is tightly correlated (27).

We also examined the impact of expression levels of a gene that regulates class II trafficking. Membrane-associated RING-CH-1 (MARCH1) is an E3 ubiquitin ligase (45) responsible for targeting cell-surface MHC class II and costimulatory CD86 molecules for degradation (46, 47). Similar to results with HLA-DRA gene expression, the association of low MARCH1 gene expression with survival was detected in a single cohort (Lenz CHOP) (data not shown). Therefore, GILT may be a better individual target for prediction of patient survival than other components of the MHC class II antigen processing and presentation pathway.

Alternatively, GILT may influence survival through additional functions outside of antigen processing. For example, GILT has been shown to alter the cellular redox state, and thus, GILT may influence survival in DLBCL through alteration of the intracellular redox environment. Superoxide is metabolized by cytosolic copper, zinc superoxide dismutase (CuZnSOD or SOD1), mitochondrial manganese SOD (MnSOD or SOD2), and extracellular SOD (EcSOD or SOD3). GILT-deficient cells have decreased expression of MnSOD and correspondingly elevated superoxide levels which may be due to increased autophagy of damaged mitochondria (48–50). GILT does not alter CuZnSOD, and reconstitution of GILT normalizes MnSOD activity (48, 49). Tome et al. found that DLBCL patients with the worst survival have alterations in the expression of genes that regulate the cellular redox environment; in particular, patients with the poorest survival had significantly lower MnSOD gene expression and no change in CuZnSOD expression (51). Thus, GILT’s alteration of cellular redox status may also contribute to the effect of GILT expression on survival in DLBCL.

To begin to identify how GILT impacts survival, we identified the cellular origin of variation in GILT expression in DLBCL tumors. We found that GILT protein expression varied widely in the DLBCL cells and that GILT protein expression in tumor cells correlated with GILT mRNA expression from the GEP studies, which was associated with patient survival. Only 47 of the DLBCL tissue specimens were linked with survival data, and this number does not provide sufficient statistical power to assess the relationship between GILT protein expression and survival using the 10% cut-off (*n* = 5 ≤ 10% GILT expression, *n* = 42 > 10% GILT expression). However, as a preliminary analysis, when comparing survival using normal GILT protein expression as a cut-off, there was a statistically significant relationship between low GILT protein expression and poor survival (*n* = 30 tumor score ≤ 2.0, *n* = 17 tumor score > 2.0, log rank test, *p* = 0.03, data not shown). Additional studies with a larger cohort are needed to draw a more definitive conclusion regarding the association of GILT protein expression with survival. Whereas GILT protein expression varied widely in DLBCL tumor cells, strong GILT protein expression was relatively conserved in tumor-infiltrating APCs. Furthermore, GILT expression in tumor-infiltrating APCs did not correlate with GILT mRNA expression. These results suggest that GILT expression is altered in tumor cells rather than a germline polymorphism resulting in low GILT expression in all cell types (52). DLBCL cells utilize mutations and deletions to inactivate the β2-microglobulin gene resulting in loss of MHC class I-restricted presentation (53). Genomic breaks in the MHC CIITA gene are found in 38% of primary mediastinal B-cell lymphomas and result in downregulation of cell-surface MHC class II expression (54). Similarly, loss of GILT expression in DLBCL cells could represent a novel mechanism of immune evasion.

In summary, these studies identify a novel association between low GILT expression and poor survival in patients with DLBCL. Loss of GILT expression in DLBCL cells may represent a mechanism of immune evasion. GILT expression provides additional information beyond established prognostic indicators. Thus, GILT merits further attention as a prognostic marker in DBLCL. Overall, we propose that antigen processing is a critical pathway in DLBCL and suggest that molecules targeting this pathway warrant investigation as potential therapeutics.

## Authors Contribution

Karen Taraszka Hastings designed the research. Hannah Phipps-Yonas and Ellen Haddock performed the research. Karen Taraszka Hastings and Hannah Phipps-Yonas analyzed and interpreted the data. Denise J. Roe and Haiyan Cui performed the statistical analysis. Noemi Sebastiao performed and Patrick S. Brunhoeber scored the immunohistochemical staining. Martin J. Deymier and Lonnie Lybarger performed the MARCH1 analysis. Wolfram Klapper provided the TMAs. Karen Taraszka Hastings wrote the manuscript along with contributions from Hannah Phipps-Yonas, Noemi Sebastiao, Patrick S. Brunhoeber, Ellen Haddock, Lonnie Lybarger, and Denise J. Roe. All authors reviewed and approved the final manuscript.

## Conflict of Interest Statement

Noemi Sebastiao and Patrick S. Brunhoeber are employees of Ventana Medical Systems who contributed to this study as part of a collaborative research agreement with no payment to either Ventana or academic investigators. The remaining authors declare no competing financial interest.
